# Utility of thermographic measurements of laterality of body surface temperature to prevent misdiagnosis of acute Wallenberg's syndrome

**DOI:** 10.1002/brb3.1040

**Published:** 2018-07-11

**Authors:** Makoto Takahashi, Akiko Shinya, Naohito Ito, Junya Ebina, Keisuke Abe, Akira Inaba, Satoshi Orimo

**Affiliations:** ^1^ Department of Neurology Kanto Central Hospital Tokyo Japan

**Keywords:** autonomic nervous system disorders, cerebrovascular diseases, neuroimaging, stroke

## Abstract

**Introduction:**

Acute Wallenberg's syndrome (WS) is sometimes misdiagnosed as a nonstroke disease including auditory vertigo, and careful neurological examination is required for a precise diagnosis. Lateral difference of body surface temperature (BST) had been reported as a symptom of WS, although further details of this symptom are currently lacking. Our aim was to investigate the laterality of BST of patients with acute WS using thermography and the usefulness of thermography to detect acute WS.

**Methods:**

Nine consecutive patients with new‐onset acute WS and nine patients with acute pontine infarction, intended for a comparison, were enrolled. Using thermography, the BST of patients was measured and initially evaluated visually. Detailed BSTs were measured using dedicated software. We examined the relationship between BST and other clinical factors, including first diagnosis, clinical symptoms, and MRI findings.

**Results:**

Four patients with WS (44.44%) were misdiagnosed with nonstroke disease and did not receive a thermography assessment at their first visit; in contrast, all acute pontine infarction patients were diagnosed with brain infarction. Eight patients with WS (89%) showed a laterality of BST at multiple sites, and three of eight patients showed a whole‐body laterality of BST; in contrast, only two pontine infarction patients showed laterality of BST at one or two sites. These lateral BST differences were easily observed visually using thermography within two minutes. The BST laterality gradually decreased over time in almost all patients with WS. The infarction size in the WS patients with whole‐body laterality of BST was craniocaudally larger than in the other patients, and the size was smallest in the patient showing no BST laterality.

**Conclusions:**

In contrast to acute pontine infarction patients, almost all patients with acute WS showed lateral BST differences, which was easily detected with thermography. Thermography may thus be a useful tool to prevent misdiagnosis of acute WS.

## 1 INTRODUCTION

Lateral medullary syndrome, also known as Wallenberg's syndrome (WS), is caused by an infarction of a wedge of the dorsal lateral medulla oblongata that results from an occlusion of the vertebral artery or posterior inferior cerebellar artery (Kim, 2003; Kameda et al., 2004). Clinical symptoms of WS include hoarseness and dysphagia, dissociated sensory disturbance, vertigo, gaze‐induced nystagmus, ataxia, and Horner's syndrome (HS), and the combination of these symptoms varies according to the anatomical structures damaged (Kim, 2003; Kameda et al., 2004; Nowak & Topka, 2006; Ogawa, Suzuki, Oishi, & Kamei, 2015; Parathan, Kannan, Chitrambalam, Aiyappan, & Deepthi, 2014). Various combinations of these symptoms have been shown in WS, although some symptoms can be weak (Kim, 2003; Kim, Lee, & Lee, 1997). In some WS cases, vertigo can be the only symptom, making it difficult to differentiate between WS and auditory vertigo (Choi, Park, Lee, & Kang, 2012), and some patients with WS are misdiagnosed with nonstroke diseases at their first visit. Lateral difference of body surface temperature (BST) has also been reported as a symptom of WS (Korpelainen, Sotaniemi, & Myllylä, 1995; Takahashi et al., 2016). This symptom is thought to result from disturbances of the sympathetic nerve tract (Korpelainen et al., 1995), although further details on this are currently lacking.

Previously, we reported the case of a patient with acute WS with HS showing bilateral discrepancy of BST resulting from an infarction‐induced disturbance of the sympathetic nerve tract (Takahashi et al., 2016). In this case, thermography easily detected the laterality of BST as although the patient was split down the middle of his body. From the thermography findings in this case, we suggest that laterality of BST helps in the identification acute patients with WS. In this study, we investigated the laterality of BST using thermography at the bed side of patients with acute WS and those with acute pontine infarction and report the usefulness of thermography to prevent the misdiagnosis of acute WS.

## 2 METHODS

We enrolled consecutive patients with acute WS and those with acute pontine infarction, for comparative purposes, who experienced a first infarction between April 2015 and December 2017. The patients with recurrent infarction were excluded. WS and pontine infarction were diagnosed according to clinical symptoms, except laterality of BST, and MRI findings.

BST was measured using a thermal camera (FLIR E5: FLIR Systems, Wilsonville, USA) in the examination room or patient's bedroom as soon as possible when making a diagnosis or suspecting WS. BST was analyzed using dedicated software (FLIR tools, http://RRID:SCR_016330, version 5.13.17214: FLIR Systems, Wilsonville, USA) at four places (the nasolabial fold on the face, palm of the hand, thoracic spine level 8–10 area at about 5 cm from the umbilicus of the torso, and the center of the dorsum of the foot) bilaterally. Laterality was defined as being positive when the following two conditions were seen concurrently: (a) The BST between the right and left sides was macroscopically different and (b) Bilateral BST discrepancy was more than 0.5°C. In the patients who showed laterality of BST, the patients with peripheral vascular stenosis, as indicated by more than 19% of laterality of branch‐ankle pulse wave velocity (baPWV; Motobe et al., 2005), which may influence the BST, were excluded.

For each patient, we also examined the mechanism of infarction, risk factors, diagnosis and plan at first visit, NIH Stroke Scale (NIHSS) score, neurological findings, and infarcted region observed on MRI, and considered the relationship of these factors with BST.

This study was approved by the Human Research Ethics Committee Institutional Review Board at Kanto Central Hospital. All the protocols were in accordance with the Ethical Principles for Medical Research involving human subjects outlined in the Declaration of Helsinki. Written informed consent was provided by all patients before enrollment.

## 3 RESULTS

### 3.1 Demographic characteristics

The demographic characteristics of all patients with WS are shown in Table 1. Nine patients with acute WS were enrolled. Patient No. 1 is a case we previously reported as “a half split‐man” who showed the laterality of body surface temperature as although he was split down the middle of his body (Takahashi et al., 2016). The mean age at diagnosis was 66.8 years. Vertebral artery dissection was the most common mechanism of infarction (five patients), next to atheromatous infarction (two patients). The most common risk factor was hypertension (six patients). Only one patient (Patient No. 6) had atrial fibrillation, and the mechanism of infarction was cardiogenic stroke.

**Table 1:** Patient characteristics and diagnosis at first visit in Wallenberg's syndrome patient

Patient			Infarction		Risk factor					First visit				
No.	Age (years)	Sex	Mechanism	Side	HT	DM	HL	Smoking	Af	First diagnosis	Diagnostician	Imaging	Imaging findings	Plan
1	51	M	Dissection	lt	+	−	−	−	−	WS	Neurologist	Brain MRI	Negative (or slight DWI high)	Admission
2	82	M	Atheroma	rt	+	−	−	−	−	Cerebellar infarction	EP →Neurologist	Brain CT	Negative	Admission
3	67	F	Dissection	lt	−	−	+	−	−	Auditory vertigo (at another hospital)	EP (at another hospital)	Brain MRI	Negative	Return home
4	56	F	Dissection	rt	+	−	−	+	−	Sensory disturbance of unknown origin	EP	Brain CT	Negative	Return home
5	66	M	Atheroma	lt	−	+	+	+	−	WS	EP →Neurologist	Brain MRI	DWI high	Admission
6	68	M	Cardioembolic	lt	+	−	−	−	+	Brainstem infarction	Neurologist	Brain MRI	Negative	Admission
7	70	M	Lacunar	lt	+	−	+	−	−	Sporadic headache	EP	Brain CT	Negative	Return home
8	69	F	Dissection	lt	+	+	+	−	−	Cerebellar infarction	EP →Neurologist	Brain MRI	Negative	Admission
9	72	M	Dissection	rt	−	+	+	−	−	Auditory vertigo	EP	Brain MRI	Negative	Admission

*Note*

Af: atrial fibrillation; DM: diabetes mellitus; DWI: diffusion‐weighted image; EP: emergency physician; F: female; HL: hyperlipidemia; HT: hypertension; lt: left; M: male; rt: right; WS: Wallenberg's syndrome.

The demographic characteristics of all pontine infarction patients are shown in Table 2. The mean age at diagnosis was 67.0 years. Lacunar infarction and branch atheromatous disease was the most common mechanism of infarction (four patients each).

**Table 2:** Pontine infarction and patient characteristics and diagnosis at first visit

Patient			Pontine infarction					First visit				
No.	Age (years)	Sex	Mechanism	Side	Extent			First diagnosis	Diagnostician	Imaging	Imaging findings	Plan
					Vertical	Horizontal	Lateral					
10	92	F	Lacunar	lt	Mid	Basis	Medial	Pontine infarction	Neurologist	Brain MRI	DWI high	Admission
11	78	F	Lacunar	rt	Up to mid	Tegmentum	Medial	Pontine infarction	Neurologist	Brain MRI	DWI high	Outpatient
12	50	M	BAD	lt	Low	Basis	Medial	Brain infarction	EP	Brain CT	Negative	Admission
13	50	M	Lacunar	lt	Mid	Tegmentum	Medial	Brain infarction	EP	Brain CT	Negative	Admission
14	75	M	BAD	rt	Mid to low	Both (mainly basis)	Medial	Pontine infarction	Neurologist	Brain MRI	DWI high	Admission
15	72	F	BAD	rt	Up	Both (mainly basis)	Medial	Pontine infarction	Neurologist	Brain MRI	DWI high	Admission
16	45	M	Dissection	lt	Low	Both	Medial	Brainstem infarction	EP→Neurologist	Brain MRI	DWI high	Admission
17	79	M	BAD	lt	Up to mid	Basis	Medial	Pontine infarction	GP→Neurologist	Brain MRI	DWI high	Admission
18	62	F	Lacunar	lt	Mid to low	Basis	Medial	Pontine infarction	EP→Neurologist	Brain MRI	DWI high	Admission

*Note*

BAD: branch atheromatous disease; both: basis + tegmentum; DWI: diffusion‐weighted image; EP: emergency physician; F: female; GP: general physician; low: lower pons; lt: left; M: male; mid: middle pons; rt: right; up: upper pons.

### 3.2 Initial diagnosis and imaging findings at the first visit

Five patients with WS were diagnosed as having brain infarction including WS at their first visit, but the other four patients with WS were misdiagnosed with nonstroke diseases (e.g., with auditory vertigo) by emergency physicians. Moreover, three of the four misdiagnosed patients were discharged (Table 1). At the first visit, all patients with WS underwent a brain CT (three patients) or a brain MRI (six patients). All but one of these findings was considered negative, although slight changes were detected in some MRI findings with retrospective observation (Table 1 and Supporting Information Figure S1).

In contrast, all patients with pontine infarction were diagnosed with brain infarction at their first visit by neurologists and nonneurologists including emergency physicians (Table 2).

### 3.3 Neurological findings at the time of WS diagnosis

The detailed symptoms at the time of WS diagnosis are shown in Table 3. The mean NIHSS score was 3.1. Truncal ataxia and dissociated sensory disturbance were present in eight patients (89%) and six patients (67%), respectively; hemiparesis was seen in one patient (11%), and tactile sensory disturbance was seen in two patients (22%). All nine patients showed HS ipsilateral to the side of the infarction, but one patient (Patient No. 2) showed only slight ptosis without anisocoria. Disturbed sweating on palpation was not evident in any of the patients.

**Table 3:** Wallenberg's syndrome patients’ symptoms

Patient No.	NIHSS	Symptoms									Horner's syndrome			
		Hemiparesis	Tactile sensory disturbance	Dissociated sensory disturbance	Limb ataxia	Truncal ataxia	Vertigo	Dysarthria	Hoarseness	Dysphasia	Pupil (mm)		Ptosis	Disturbed sweating on palpation
											rt	lt		
1	2	−	+	+	+	+	+	−	−	−	4	3	lt	−
2	6	+	−	+	+	+	+	−	−	−	3	4	rt	−
3	5	−	+	+	−	+	+	−	+	+	3.5	3	lt	−
4	1	−	−	+	−	+	−	−	−	−	3	4	rt	−
5	3	−	−	+	+	+	+	−	−	−	4	3	lt	−
6	2	−	−	−	+	+	−	+	−	−	3	2.5	lt	−
7	3	−	−	+	−	−	−	+	+	+	4	3	lt	−
8	3	−	−	−	+	+	−	−	−	−	4	3	lt	−
9	3	−	−	−	+	+	+	−	+	+	4	4	rt	−

*Note*

lt: left; rt: right.

The details of symptoms at the time of pontine infarction diagnosis are shown in Table 4. The mean NIHSS score was 3.4. Dysarthria was present in seven patients (78%), hemiparesis was seen in six patients (67%), and tactile sensory disturbance was seen in three patients (33%). No patient exhibited Horner's syndrome.

**Table 4:** Pontine infarction patients’ symptoms

Patient No.	NIHSS	Symptoms										Horner's syndrome			
		Laterality of BST	Hemiparesis	Tactile sensory disturbance	Dissociated sensory disturbance	Limb ataxia	Truncal ataxia	Vertigo	Dysarthria	Hoarseness	Dysphasia	Pupil (mm)		Ptosis	Disturbed sweating on palpation
												rt	lt		
10	3	‐	+	‐	‐	+	‐	‐	+	‐	‐	‐	‐	‐	‐
11	0	‐	‐	‐	‐	‐	‐	‐	‐	‐	‐	‐	‐	‐	‐
12	5	‐	+	‐	‐	+	‐	‐	+	‐	‐	‐	‐	‐	‐
13	1	‐	‐	+	‐	‐	‐	‐	‐	‐	‐	‐	‐	‐	‐
14	3	+	+	‐	‐	‐	‐	‐	+	‐	‐	‐	‐	‐	‐
15	3	‐	+	‐	‐	‐	‐	‐	+	‐	‐	‐	‐	‐	‐
16	7	+	+	+	‐	‐	‐	+	+	‐	‐	‐	‐	‐	‐
17	7	‐	+	‐	‐	‐	‐	‐	+	‐	‐	‐	‐	‐	‐
18	2	‐	‐	+	‐	‐	‐	‐	+	‐	‐	‐	‐	‐	‐

*Note*

BST: body surface temperature; lt: left; rt: right.

### 3.4 Laterality of body surface temperature

**Figure 1:**
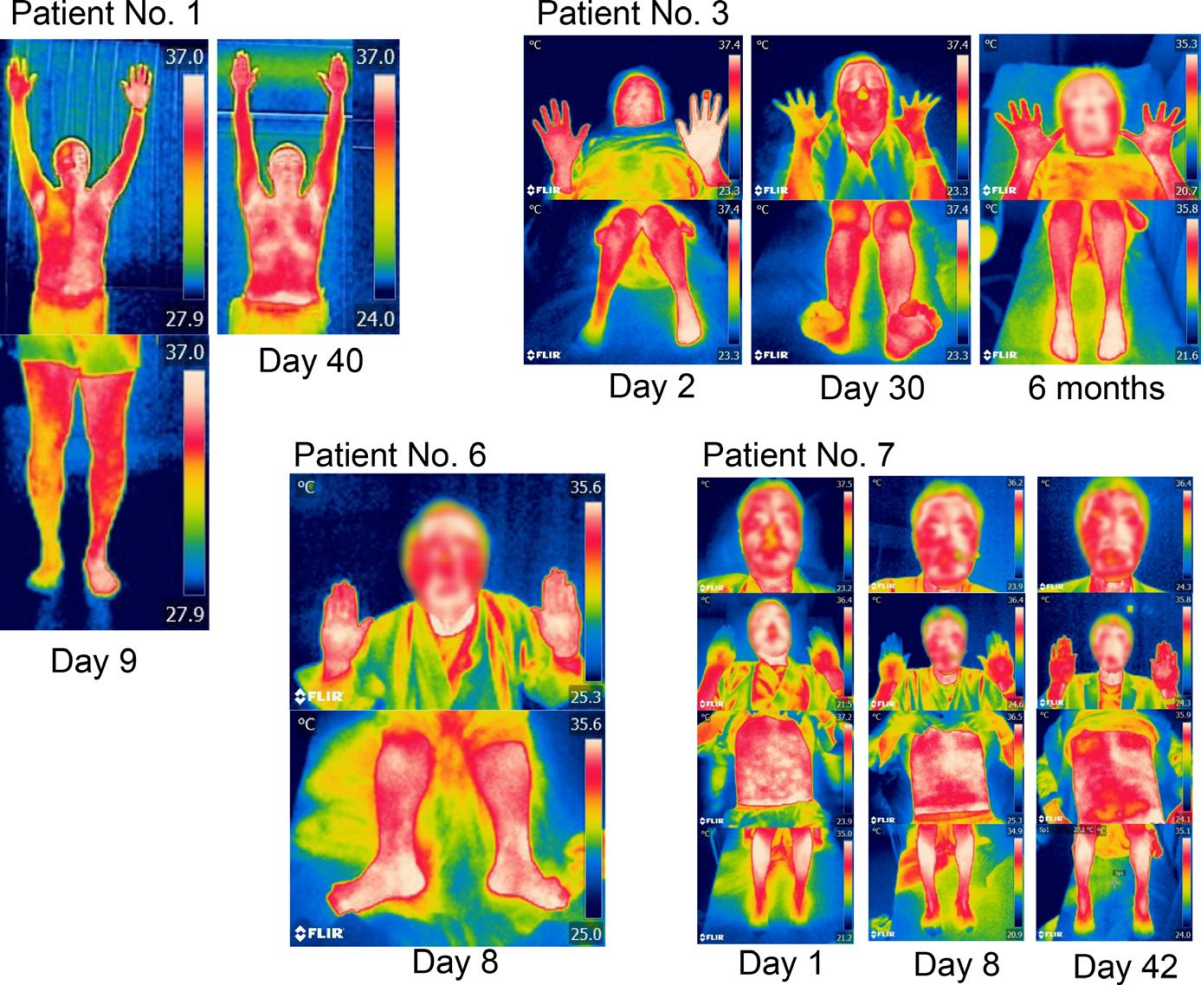
Pictures taken by a thermographic camera in typical patients with WS. Patient No. 1: The body surface temperature (BST) shows a whole‐body lateral discrepancy, split down the middle of his body. This laterality of BST was diminished at 40 days after infarction. Patient No. 3: Her BST at the upper and lower extremities showed a laterality that decreased gradually over time. Patient No. 6: Laterality of BST was not apparent in the acute stage. Patient No. 7: The laterality of BST was seen at all regions except the lower legs. The degree of laterality of BST fluctuated over time (The faces of the patients are obscured by a mosaic for personal information protection)

Detailed thermography results of patients with WS are shown in Table 5. The mean duration from disease onset to taking the thermographic picture was 3.6 days (range: 1–9 days). In all patients with WS, it took within 2 min to take the thermographic picture of the whole body and visualize the laterality of BST. All but one patient with WS (89%) showed laterality of BST (Figure 1 and Table 5). In all these eight patients with WS, the warmer BST side was ipsilateral to the infarction, as was HS. While the region showing laterality of BST differed in each case, all these eight patients with WS showed BST at multiple sites. Three patients showed whole‐body laterality of BST, and three patients showed laterality of BST in all but one region (face, lower limb, and upper limb). Two patients showed lateralized BST at the upper and lower extremities. Seven of the eight patients with WS who had laterality of BST showed the most discrepant BST in the lower limbs, which showed a discrepancy of up to 9.5°C. The laterality of BST in the lower extremities could be detected with palpation in almost every patient who showed laterality of BST in the foot.

**Table 5:** Detailed thermography results in Wallenberg's syndrome patients

	Thermography											
PatientNo.	Timing (days)	Visual evaluation	Temperature (°C)								Follow up	
			Face		Body		Upper Limb		Lower limb		Continuance	Disappearance
			rt	lt	rt	lt	rt	lt	rt	lt		
1	9	〇	34.8	36.2	34.5	35.5	34.8	36	32.6	35.6	—	40 d
2	1	〇	35.2	34.2	36.4	35.3	35.7	27.5	33.9	25.4	22 d	n.d.
3	2	〇	37.2	37.5	37.5	37.5	34.3	36.7	27.7	37.2	30 d	6 m
4	2	〇	35.9	35.5	35.8	35.1	36.6	34.7	34.6	29.8	20 d	5 m
5	2	〇	37.5	37.6	36.5	37.1	31.1	33	28.7	31.1	14 d	n.d.
6	8	×	35	35.1	35.4	35.3	35.2	35.3	35.4	35.5	—	—
7	1	〇	35.2	36.6	36.5	37.1	31.3	32.8	30.4	30.1	42 d	f.u.
8	2	〇	36.2	36.8	35.9	37.1	36.2	36.6	32.9	35.7	4 m	f.u.
9	5	〇	37.6	36.7	35.8	35.2	37	35.5	35.8	33.7	40 d	f.u.

*Note*

continuance: the time showing the continuance of laterality of body surface temperature; d: days; disappearance: the time showing the disappearance of body surface temperature; lt: left; m: months; rt: right.

Gray shades show the presence of lateral differences of body surface temperature and darker gray shades show the colder side of BST.

**Figure 2:**
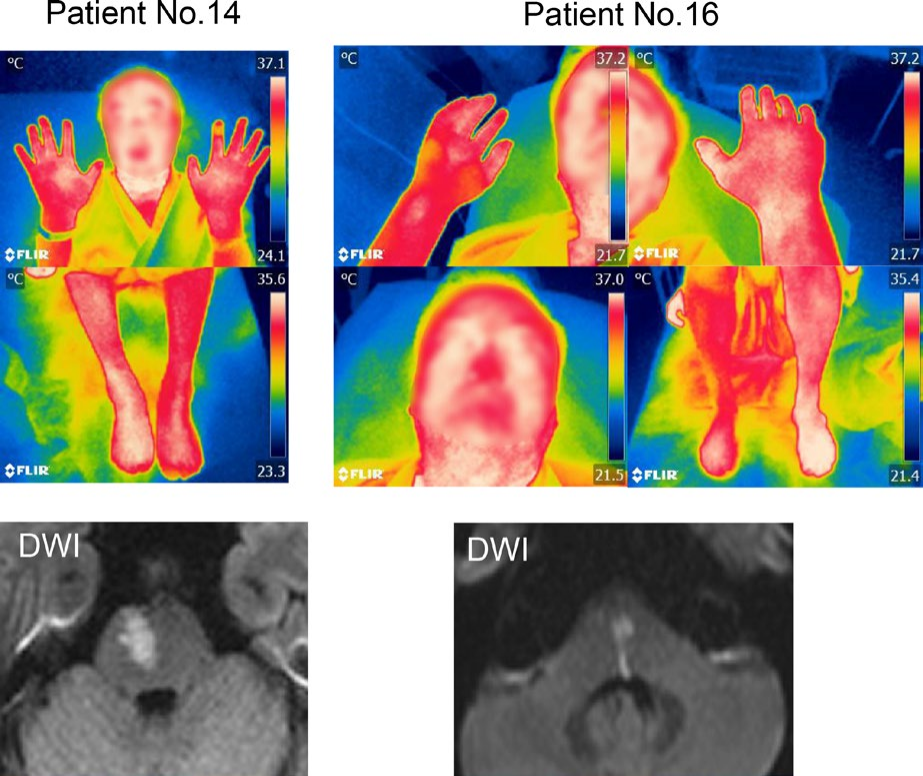
Picture taken by thermographic camera and MRI findings in pontine infarction patients with laterality of BST. Two patients with lower pontine infarction showed the laterality of BST only at one or two of their extremities (The patients’ faces are obscured by a mosaic for personal information protection)

In the nine acute pontine infarction patients, only two patients (Patient Nos. 14 and 16) showed laterality of BST (Table 4 and Figure 2). One patient showed laterality of BST only at the foot (Patient No. 14) and the other (Patient No. 16) at the hand and foot. The warmer BST side of these two patients was also ipsilateral to the infarction as was the case with patients with WS.

The time course of the laterality of BST was followed over 14 days in all eight patients with WS who showed a lateralized BST, and over 30 days in six patients with WS (Table 5). The degree of discrepancy of BST gradually decreased with time in almost all patients with WS; however, a few patients with WS showed fluctuations of discrepancy of BST at the acute to subacute stages (Figure 1). In three of these eight patients with WS, the laterality of BST disappeared at 40 days, 5 months, and 6 months. Another three of these eight patients with WS showed laterality of BST at their most recent visit and followed at 40 days, 42 days, and 4 months. It was not possible to follow the other two patients after 14 days and 22 days after onset.

### 3.5 Brain MRI findings and BST

**Figure 3:**
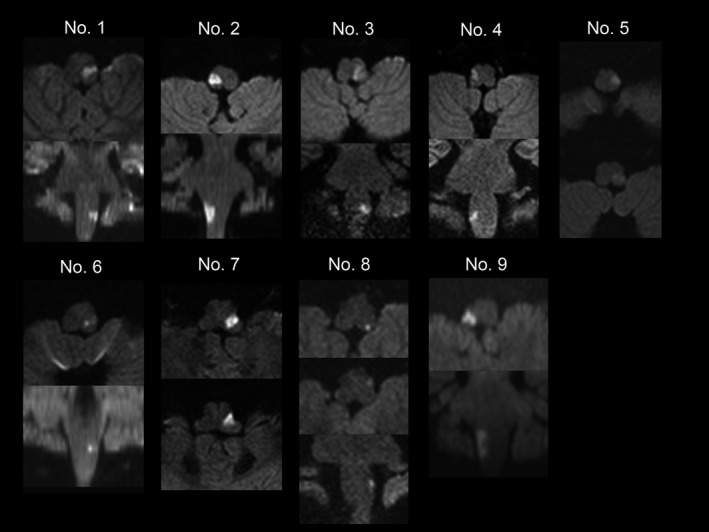
MRI findings at the medulla. The DWI high‐intensity lesion in Patients No. 1, 2, and 9, who showed a whole‐body laterality of BST, was craniocaudally longer than all other patients. In contrast, DWI high‐intensity lesion in Patient No. 6, who showed no laterality of BST, was the smallest of all patients

Although the first brain MRI findings in four patients with WS were initially considered negative, all nine patients with WS showed DWI hyperintensity at the lateral medulla over the course of the disease (Table 1, Figure 3, and Supporting Information Figure S1). The infarct size of patients No. 1, No. 2, and No. 9, who showed a whole‐body laterality of BST, was craniocaudally longer than other cases and was smallest in Patient No. 6, who showed no laterality of BST.

In all pontine infarction patients, the first brain MRI showed abnormal DWI high‐intensity lesion (Table 2). Four patients had infarction in the basis, two in the tegmentum, and three in both (Table 2). All pontine infarction patients had infarction in the medial portion, and no patient showed lateral pontine infarction. The patients with lower pontine infarction with an anteroposterior width (Patient Nos. 14 and No. 16) showed laterality of BST (Figure 2).

## 4 DISCUSSION

In this research, we focused on the laterality of BST in acute patients with WS compared to pontine infarction patients and observed two important findings about clinical aspects of WS.

The first important finding was that four of the nine patients with WS were misdiagnosed as having a nonstroke disease at their first visit, three of which were discharged; in contrast, all pontine infarction patients were diagnosed with stroke. According to our results, we can suggest two reasons for these misdiagnoses of WS. First, the characteristic symptoms of WS are rather mild; it has been reported that the severity of brainstem infarction is relatively low and prognosis is good (Glass et al., 2002; Akhtar et al., 2009; Fitzek et al., 2001). Indeed, the mean NIHSS score of our patients with WS was also as low as 3.1, and some patients came to the outpatient clinic by foot and with only minor complaints. However, mild symptoms were also seen in the patients with pontine infarction, with a NIHSS score of 3.4. Another unique symptomatic characteristic of patients with WS causing misdiagnosis was the rarity of hemiparesis and tactile sensory disturbance, which tend to be present in patients with other brainstem infarctions and supratentorial infarction; in contrast, they show HS and dissociated sensory disturbance (Kim, 2003; Kameda et al., 2004; Parathan et al., 2014; Korpelainen et al., 1995). In our study, hemiparesis was observed in only one patient with WS, and tactile sensory disturbance in only two patients with WS. In contrast, eight of the nine pontine infarction patients showed hemiparesis and/or tactile sensory disturbance. These symptomatic characteristics of WS mean that it is difficult to infer the presence of an infarction at the first visit, especially for nonneurologists. Second, misdiagnosis may be a result of the delay of appearance of high‐intensity DWI signal in WS. Indeed, it has been reported that high‐intensity DWI signal on brainstem infarctions has a delayed appearance in some cases (Oppenheim et al., 2000; Tsuyusaki et al., 2014). Similarly, almost all our cases showed no DWI high intensity at their first visit.

The second important finding is that almost all patients with WS showed the laterality of BST in the acute stage and a thermography can easily, rapidly, and noninvasively detect the laterality of BST; thermography can be a useful tool to diagnose acute WS. The laterality of BST at multiple sites on the same side was the second most common symptom in patients with acute WS next to HS. Because the degree of lateral discrepancy of BST gradually decreased in almost all patients, the presence of large lateral differences of BST at multiple sites could be indicative of acute‐stage WS.

The degree of lateral discrepancy of BST was greatest at the foot region in seven of the eight patients and could be detected with palpation in almost all patients. However, most of these patients might not notice the laterality of BST by themselves, because thermal threshold in the lower limb is lower than in the upper limb (Hafner et al., 2015) and a significant proportion of patients with WS have dissociated sensory disturbance including temperature sensory disturbance (Kim, 2003; Kameda et al., 2004). When WS is suspected, touching and feeling the discrepancy of BST in the lower extremities on palpation may be a good first step to the diagnosis of WS if a thermography assessment is not possible.

BST is influenced by both, vascular factors such as arterial stenosis and nerve factors such as autonomic nerve disturbance. In the present study, the mechanism of the appearance of the laterality of BST in WS is thought to be an autonomic disturbance rather than vascular stenosis, because of the following three reasons: (a) patients who were suspected to have vascular stenosis according to baPWV results were excluded, (b) the laterality of BST was shown at multiple sites of the same side in all patients who showed laterality of BST, and (c) the laterality of BST decreased over the course of the disease. Autonomic thermoregulatory effector mechanisms consist of shivering and nonshivering thermogenesis for heat production and cutaneous vasomotor response and sweating for heat dissipation (Low, 1997). Because heat production mechanisms affect the whole body, disturbance of this mechanism cannot be the origin of the laterality of BST. Considering that the warmer side of all patients showing the laterality of BST was ipsilateral to the side of the infarction, we suggest that the cause of the laterality of BST in patients with WS is a disturbance of the connecting pathway of sweating and skin blood flow descending from the lateral brainstem, including the ventrolateral medulla (Low, 1997), which is close to the HS pathway (Crevits, D'Herde, & Deblaere, 2004). This disturbed sweating and disturbed vasoconstriction is likely to increase the BST on the ipsilateral side. Based on the mechanism of laterality of BST as noted above, not only the WS but also other infarctions could show the laterality of BST. In fact, the patients with pontine infarction in this study showed the laterality of BST, however, a small number of patients with lower pontine infarction with an anteroposterior width showed the laterality of BST only at one or two of their extremities. This characteristic difference of laterality of BST between WS and pontine infarction may be due to the difference of contiguity of the connecting pathways. Because the area controlling the sympathetic skin vasomotor fiber is thought to be smaller in the medulla than in the pons (Nakazato, Shimazu, Tamura, & Hamaguchi, 1995), infarction in the medulla may cause laterality of BST with a higher frequency and in a broader body portion.

Only one patient with WS (Patient No. 6) showed no laterality of BST in this research. This might be due to the timing of observation; the thermography was conducted later, 8 days after the infarction. However, the fact that other symptoms, including HS, were observed in this patient at that moment, as well as the fact that laterality of BST of other patients was shown at least 14 days after infarction, indicate that laterality of BST did not exist in this patient. While the clinical background and symptoms of this patient were not notably different from those of the other enrolled patients, the mechanism of infarction was cardioembolic stroke and atrial fibrillation was noted; furthermore, this patient had the smallest infarct size of all patients. In contrast, the infarct sizes of the three patients showing whole‐body laterality of BST were craniocaudally longer than those of the other patients. Thus, we can suggest that the longitudinal infarct size is related to the presence and the region of the laterality of BST.

The study has some limitations. First, only a small number of patients participated in the study; the rarity of brainstem infarction, including WS, meant that only nine patients with WS and nine with pontine infarction, included for comparative purposes, were enrolled. However, selection bias was eliminated because we only enrolled consecutive patients who fulfilled the inclusion criteria. The second limitation is that thermography was conducted at different time points in each patient. Because the degree of lateral discrepancy of BST was found to decrease over time, observational delay may render the degree of lateral discrepancy of BST small.

## 5 CONCLUSION

In contrast to the patients with acute pontine infarction, almost all of patients with WS in this study showed a laterality of BST in the acute stage, which was easily detected using thermography. It is sometimes difficult to diagnose WS in the acute stage compare to the other brain infarction because of the symptomatic characteristics and delayed appearance of abnormalities in brain MRI scans; thus, thermography may be a useful tool to assist with WS diagnosis. Our results suggest that we should check the laterality of BST before diagnosis and consider the diagnostic value of thermography. We hope to conduct further multicenter studies on BST, not only in patients with brainstem infarctions including WS but also in those with other type of stroke and dizziness.
