# Supplementary figures and images for: Utility of thermographic measurements of laterality of body surface temperature to prevent misdiagnosis of acute Wallenberg's syndrome
Source: Brain Behav. 2018 Jul 11;8(8):e01040. doi: 10.1002/brb3.1040 (PMC6085920; doi:10.1002/brb3.1040)

Patient No. 1

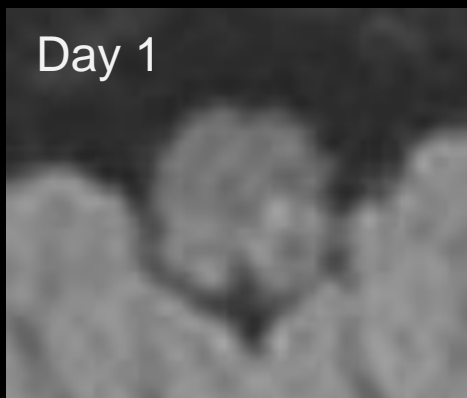

Patient No. 6

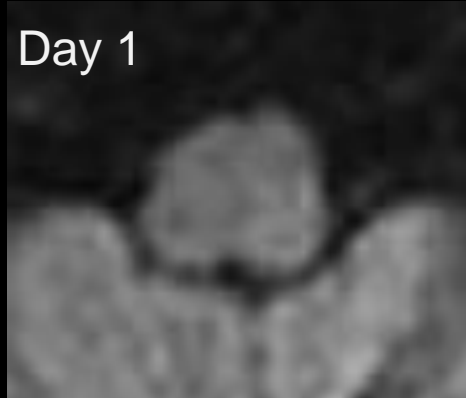

Patient No. 7

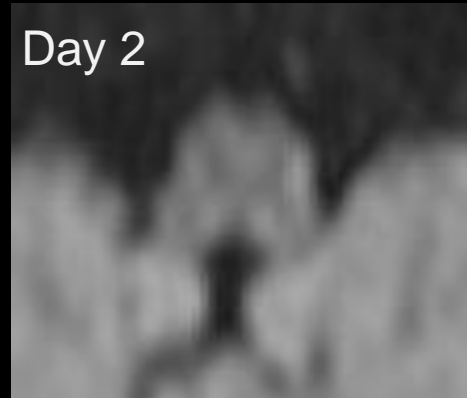

Patient No. 9

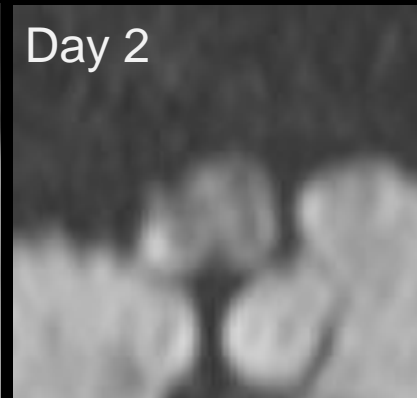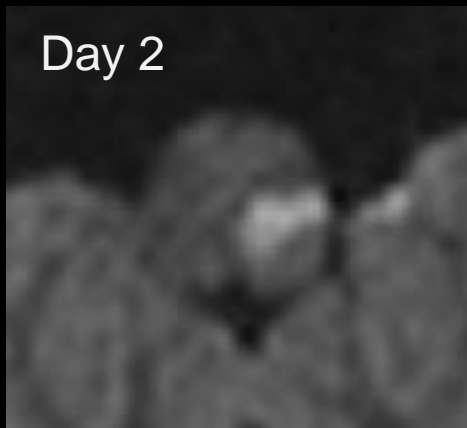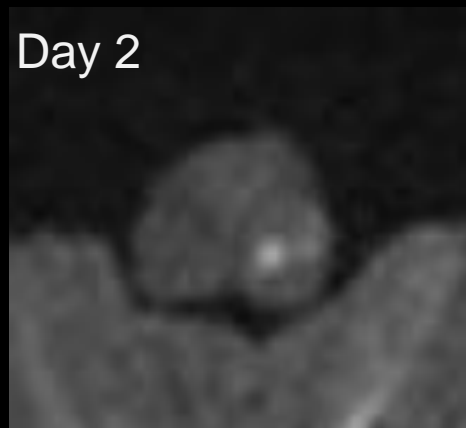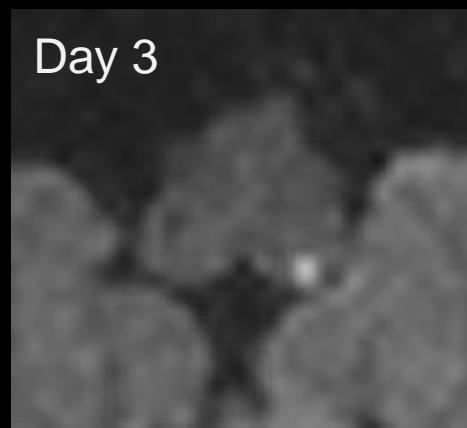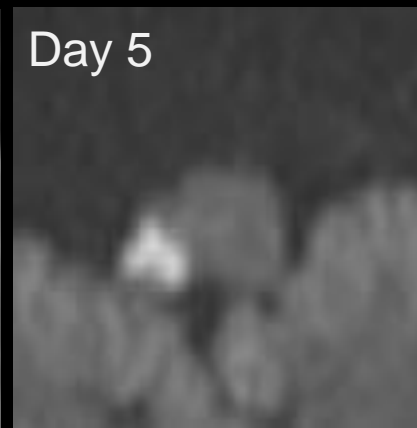

Supplement: Supplementary file 1 [file BRB3-8-e01040-s001.pdf]
