# Supplementary material for: Utility of thermographic measurements of laterality of body surface temperature to prevent misdiagnosis of acute Wallenberg's syndrome
Source: Brain Behav. 2018 Jul 11;8(8):e01040. doi: 10.1002/brb3.1040 (PMC6085920; doi:10.1002/brb3.1040)
Supplement: Supplementary file 2 [file BRB3-8-e01040-s002.docx]

**Supplementary information**

Supplementary Figure 1. Change of MRI diffusion-weighted image findings in the medulla between first and second visits.
